# Changing research trends in seed responses to stresses: a bibliometric analysis over the last 50 years

**DOI:** 10.3389/fpls.2025.1691250

**Published:** 2026-01-27

**Authors:** Zhouli Liu, Lin Qi, Benyang Hu, Yuchen Zhao, Hetong Wang, Nan Zhang, Xiangbo Duan, Binglun Li, Mingran Xin, Sihui Zhong, Hengyu Liu

**Affiliations:** 1 College of Life Science and Engineering, Shenyang University, Shenyang, China; 2 Northeast Geological S&T Innovation Center of China Geological Survey, Shenyang, China; 3 Key Laboratory of Black Soil Evolution and Ecological Effect, Ministry of Natural Resources, Shenyang, China; 4 Key Laboratory of Ecological Restoration of Regional Contaminated Environment, Ministry of Education, College of Environment, Shenyang University, Shenyang, China; 5 Institute of Applied Ecology, Chinese Academy of Sciences, Shenyang, China

**Keywords:** development dynamics, environmental stress, plant seeds, research trends, visualized analysis

## Abstract

**Introduction:**

Abiotic stresses (e.g., drought, salinity, heavy metals) intensified by global environmental changes threaten plant seed germination, seedling establishment, and population persistence. Elucidating the spatio-temporal dynamics and adaptive mechanisms of seed stress responses is critical for ecological conservation and stress-resistant crop breeding, yet long-term global and regional research trends lack systematic integration.

**Methods:**

A bibliometric analysis was conducted using CiteSpace v.6.4.R1 to process 15,627 literature records (9,042 from Web of Science; 6,585 from CNKI) spanning 1975–2024, focusing on publication dynamics, cooperation networks, intellectual base, research hotspots, and emerging frontiers.

**Results:**

Seed-stress research evolved through three stages (initial exploration, rapid growth, steady breakthrough), with WOS and CNKI seeing annual increases of 684 and 453 articles post-2020. China led in WOS publication volume, but cooperation networks showed low connectivity. International high-cited literature centered on salinity/oxidative stress (80% reviews), while domestic research focused on staple crop stress responses and practical indicators. Post-2020, international frontiers leaned toward nanomaterials and signal transduction, and domestic frontiers prioritized cadmium pollution control and germination regulation.

**Discussion:**

Global research presents a diverse, integrated landscape, while domestic research exhibits strong application orientation with relative fragmentation. Future research should integrate basic mechanisms with practical needs, strengthen interdisciplinary/international collaboration, and focus on combined stress adaptation and green regulatory technologies, providing theoretical and technical support for enhancing plant stress resistance and ecological security.

## 1 Introduction

Recently, with the population growing and human activities intensifying, environmental stress is becoming increasingly severe, posing significant challenges to ecosystems and human well-being (Cai et al., 2025). Environmental stress has long been recognized as a critical factor shaping plant adaptation and survival across diverse ecosystems (Jalal et al., 2025; Ma et al., 2025). Over the past 50 years, increasing global environmental stresses, including abiotic factors such as climate variability, drought, salinity, extreme temperatures, waterlogging, and heavy metal pollution, has posed significant challenges to plant reproduction and survival (Li et al., 2025a, 2025; Miao et al., 2025; Senthamil et al., 2025; Song et al., 2025; Zhakypbek et al., 2025). These stressors can alter seed development, dormancy mechanisms, germination timing, and seedling establishment, thereby affecting the reproductive success and population dynamics of plant species (Alam et al., 2025; Chen et al., 2025a, 2025; Naaz et al., 2025). For instance, drought stress can induce seed dormancy to delay germination until more favorable conditions arise, while salinity may inhibit germination altogether (Guo et al., 2024; Han et al., 2025). Heavy metals have also been shown to reduce seed germination rates and negatively affect the initial growth of seedlings (Uslu et al., 2025). Moreover, climate change and other anthropogenic activities are exacerbating the frequency and intensity of these environmental stressors, making it imperative to understand the adaptive mechanisms of plant seeds (Chen et al., 2020; Kuroda and Sawada, 2022).

Plants have evolved a remarkable array of strategies to survive and reproduce in diverse and often challenging environments (Alam et al., 2025; Cai et al., 2025; Miao et al., 2025; Naaz et al., 2025; Zhakypbek et al., 2025). One of the most critical stages in the plant life cycle is the seed stage, which serves as the primary means of dispersal and the initial phase of establishment for the next generation (Chen et al., 2020; Guo et al., 2024). Plant seeds exhibit remarkable sensitivity to a wide array of environmental cues, including abiotic factors such as temperature fluctuations, light availability, moisture levels, and soil chemistry (Chen et al., 2020; Kuroda and Sawada, 2022; Guo et al., 2024; Moreira et al., 2024; Chen et al., 2025a; Liu et al., 2025a). These environmental cues can significantly affect their germination, dispersal, viability, and the establishment of new generations. Understanding how plant seeds respond to environmental stress is crucial for predicting plant population dynamics, species distribution, and ecosystem resilience in the face of global environmental change (Safavi et al., 2023; Wang and Zhang, 2023). Presently, most existing research focuses on short-term responses or specific stressors, often neglecting the cumulative and interactive effects of multiple stressors over time (Chen et al., 2020; Kuroda and Sawada, 2022; Safavi et al., 2023; Wang et al., 2023; Guo et al., 2024; Moreira et al., 2024; Chen et al., 2025a; Liu et al., 2025b; Uslu et al., 2025). This gap in knowledge hampers our ability to fully understand how plant seeds have adapted to environmental changes over the past decades and how they might respond to future stressors. Grasping the spatio-temporal dynamics in plant seed characteristics in response to environmental stresses is essential for predicting plant population resilience and shaping conservation strategies in a changing world.

As a leading bibliometric visualization tool, CiteSpace has garnered significant attention in recent years (Xu et al., 2022; Ge et al., 2024; Wen et al., 2024; Ding et al., 2025; Liu et al., 2025c). It provides essential data support for quantitative analysis, enabling researchers to explore future research directions by analyzing the growth of publications in specific areas (Hu et al., 2019; Wang et al., 2023; Yuan and Lai, 2023; Wu et al., 2024a). CiteSpace has been successfully applied to study the dynamics of vegetation cover and ecosystem responses to environmental change, offering insights into the adaptive mechanisms of plants (Li et al., 2023; Chen et al., 2024a; Ge et al., 2024; Hu et al., 2024; Lei et al., 2025; Yang et al., 2025). However, despite these technological advancements, comprehensive studies on the spatio-temporal dynamics of plant seed characteristics over long periods remain limited. Therefore, this study utilized CiteSpace v.6.4.R1 software to synthesize and analyze data from the Web of Science (WOS) and the China National Knowledge Infrastructure (CNKI) databases, with the analysis covering the period from 1975 to 2024. The primary objectives of this study are to: (1) explore the spatio-temporal distribution of research on plant seed characteristics in response to environmental stresses; (2) identify the key research hotspots and development trends in the field of “seed-stress” over the past 50 years; and (3) provide valuable insights into the underlying mechanisms of plant resilience. This study will be helpful for scholars to gain a quantitative and intuitive grasp of the current state of research and to track the dynamic frontiers in the field of “seed-stress.” Additionally, it will offer a holistic understanding of plant stress resistance and provide valuable insights for ecological management and conservation.

## 2 Materials and methods

### 2.1 Data sources

**Figure 1:**
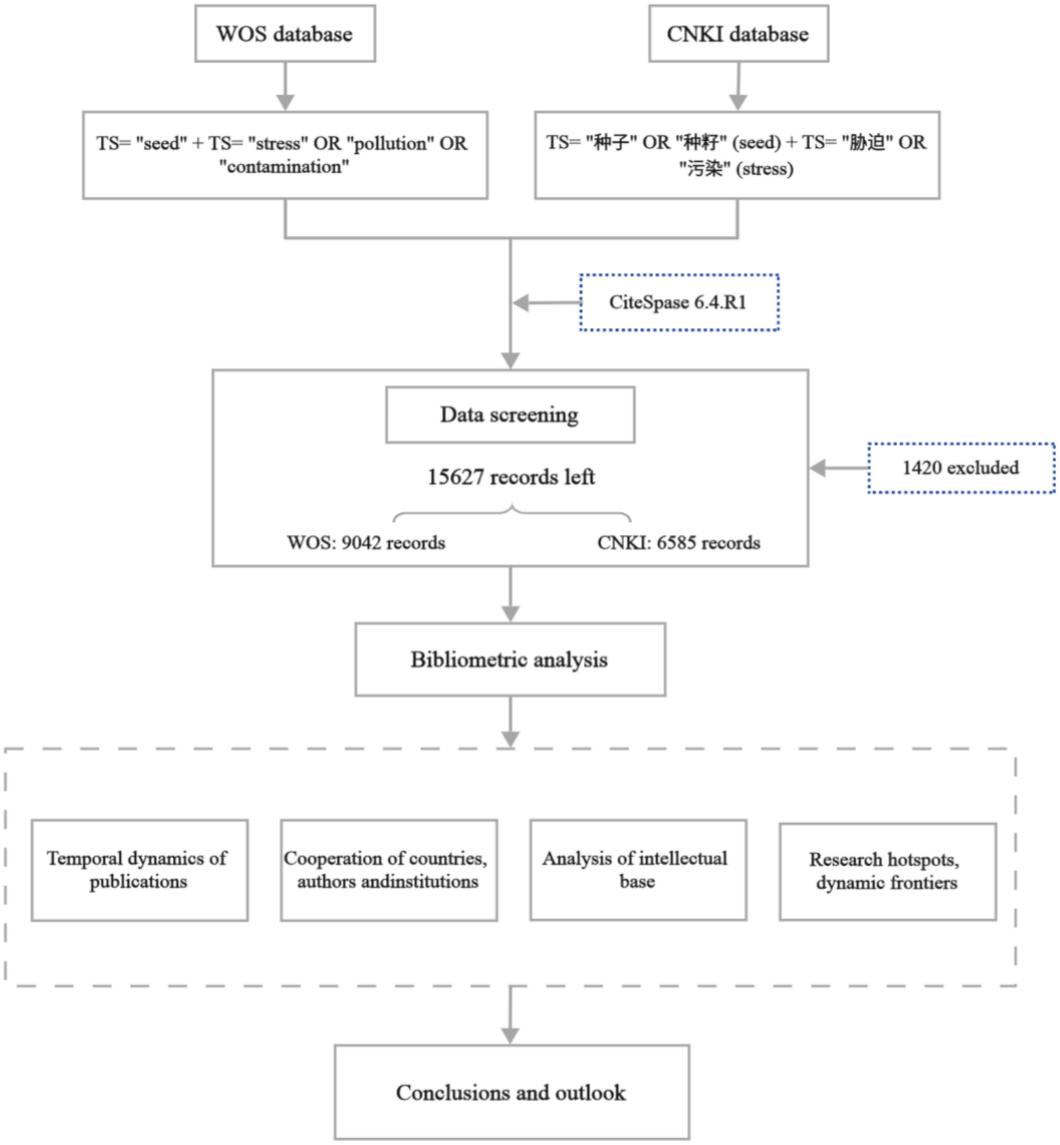
The flowchart of the study. Note: WOS denotes the Web of Science database; CNKI denotes the China National Knowledge Infrastructure database; TS denotes topic.

The dataset employed in this study was retrieved from two reputable repositories: the Web of Science (WOS) and the China National Knowledge Infrastructure (CNKI). These databases are renowned for their extensive compilation of peer-reviewed scholarly publications, spanning diverse disciplines and regions globally. Their robustness and wide coverage collectively underpin the robustness and authoritative nature of the data utilized in this research. The temporal scope of this study encompasses the period from January 1975 to December 2024. On January 2025, a comprehensive search was conducted to retrieve scholarly publications related to the topic of “seed and stress” from the WOS and CNKI databases. In the WOS database, the search query was formulated as follows: (TS= “seed” + TS= “stress” OR “pollution” OR “contamination”). In the CNKI database, the search query was formulated as follows: subject=[“种子” OR “种籽” (seed) + TS= “胁迫” OR “污染” (stress)]. After meticulous review and screening, a total of 1420 irrelevant or duplicate records were excluded. This process resulted in the retention of 15627 relevant literature records, comprising 9042 from the WOS database and 6585 from the CNKI database, all pertaining to the topic of “seed-stress.” These curated records will serve as the fundamental dataset for subsequent analysis. The flowchart of this study, which outlines the steps of bibliometric analysis, is presented in **Figure 1**.

This systematic review follows the core principles of the PRISMA guidelines. A detailed retrieval and screening flowchart (**Figure 1**) is provided, including the number of records identified, excluded, and included. The retrieval strategy (search terms, databases, time range) is explicitly described to ensure transparency and reproducibility. Given that this study focuses on bibliometric synthesis (analyzing research trends and knowledge structures) rather than empirical evidence integration, we adopt CiteSpace-based visualization synthesis, which is consistent with the reporting standards for bibliometric systematic reviews.

### 2.2 Analysis method

Given its intuitive visualization capabilities and robust data processing functions, CiteSpace has become a powerful tool for analyzing and visualizing scientific literature (Liu et al., 2022; Chen et al., 2024b; Wang et al., 2025a). It not only simplifies complex data into understandable graphical representations but also provides detailed insights into research trends and key knowledge nodes (Chen et al., 2022; Wu et al., 2024a; Qu et al., 2025). These features have enabled CiteSpace to find extensive application across diverse research domains, including environmental pollution and plant science (Zhang et al., 2020; Chen et al., 2024c; Jiang et al., 2024; Liu et al., 2025a). In this study, CiteSpace software (version 6.4.R1) was employed to conduct a visual bibliometric analysis based on the curated literature records obtained from the WOS and CNKI databases. For the analysis of publication growth trends and major publication outlets, data processed through CiteSpace was utilized. This data was subsequently refined and organized to generate visual graphs for bibliometric scrutiny. Additionally, CiteSpace was used to graphically depict the distribution of authors, countries, research institutions, and keywords since 1975. CiteSpace underscores the utilization of connecting lines to delineate the significance of distinct subject areas and to scrutinize the clustering interconnections among diverse nodes (Qin et al., 2021; Li et al., 2022a; Wei et al., 2024; Qi et al., 2025). Consequently, the software is adept at accurately elucidating the central facets of research topics pertinent to “seed-stress”. Moreover, the general publication trends were analyzed using Microsoft Office Excel 2020 and Origin 2022 to generate the corresponding charts.

## 3 Results

### 3.1 Temporal dynamics of publications on “seed-stress” research

**Figure 2:**
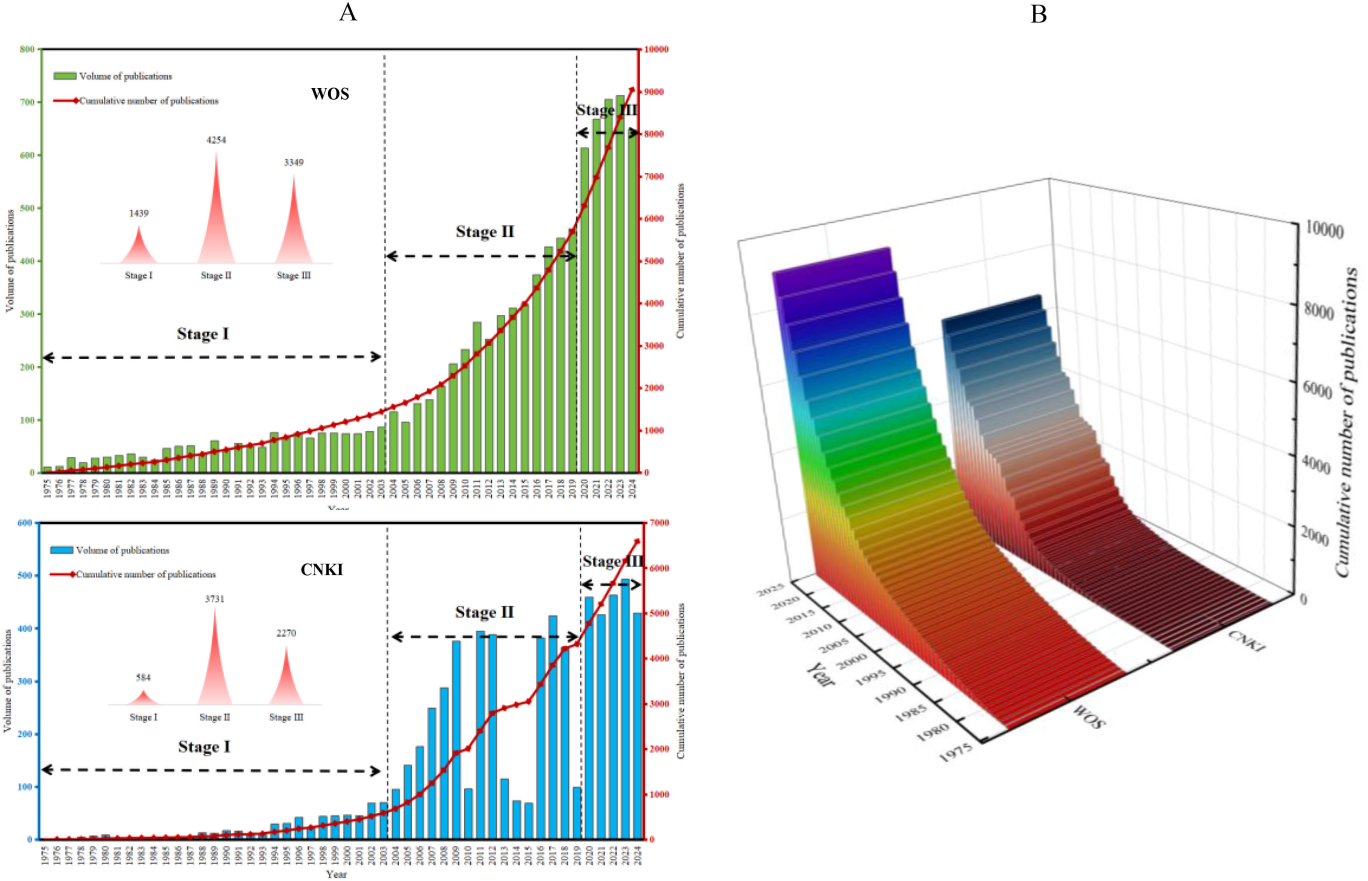
Annual publication counts, phase-specific cumulative volumes **(A)**, and inter-database cumulative trend comparison of “seed-stress” research **(B)** (1975–2024). Note: WOS denotes the Web of Science database; CNKI denotes the China National Knowledge Infrastructure database.

The analysis of scholarly publications provides valuable insights into the development and trends within a specific research domain (Li et al., 2022b). By examining the volume, trajectory, and composition of scholarly publications, researchers can gain insights into the annual publication volume, cumulative publication volume, and annual average increase, thereby forecasting future trends in scholarly output within their field of study (Liu et al., 2024a). In this study, a total of 15,627 relevant literatures on “seed-stress” were analyzed, including 9,042 from the WOS database and 6,585 from the CNKI database. The annual and cumulative publication volumes during 1975–2024 (**Figure 2**) showed that research in both databases followed three distinct stages: Stage I (Initial Exploration Phase, 1975–2003): WOS recorded 1,430 publications, while CNKI had 584 publications; Stage II (Rapid Growth Phase, 2004–2019): WOS publications increased to 4,254, and CNKI to 3,731, narrowing the gap between the two databases; Stage III (Steady Breakthrough Phase, 2020–2024): WOS published 3,349 articles, and CNKI 2,270. The global average annual increase in WOS was 684 articles, while that in CNKI was 453 articles.

The cumulative publication volume (**Figure 2**) showed that the WOS database (9,042 literatures) was 37.31% higher than the CNKI database (6,585 literatures). The cumulative number of literatures in the WOS database showed a continuous and stable upward trend, indicating that “seed-stress” research remained active globally. Although the CNKI database also showed an overall growth trend, there were slight fluctuations, reflecting that domestic research was affected by factors such as policies and funding investment, resulting in slight variations in the pace of development.

### 3.2 Spatial dynamics of publications on “seed-stress” research

#### 3.2.1 Country cooperation networks

**Figure 3:**
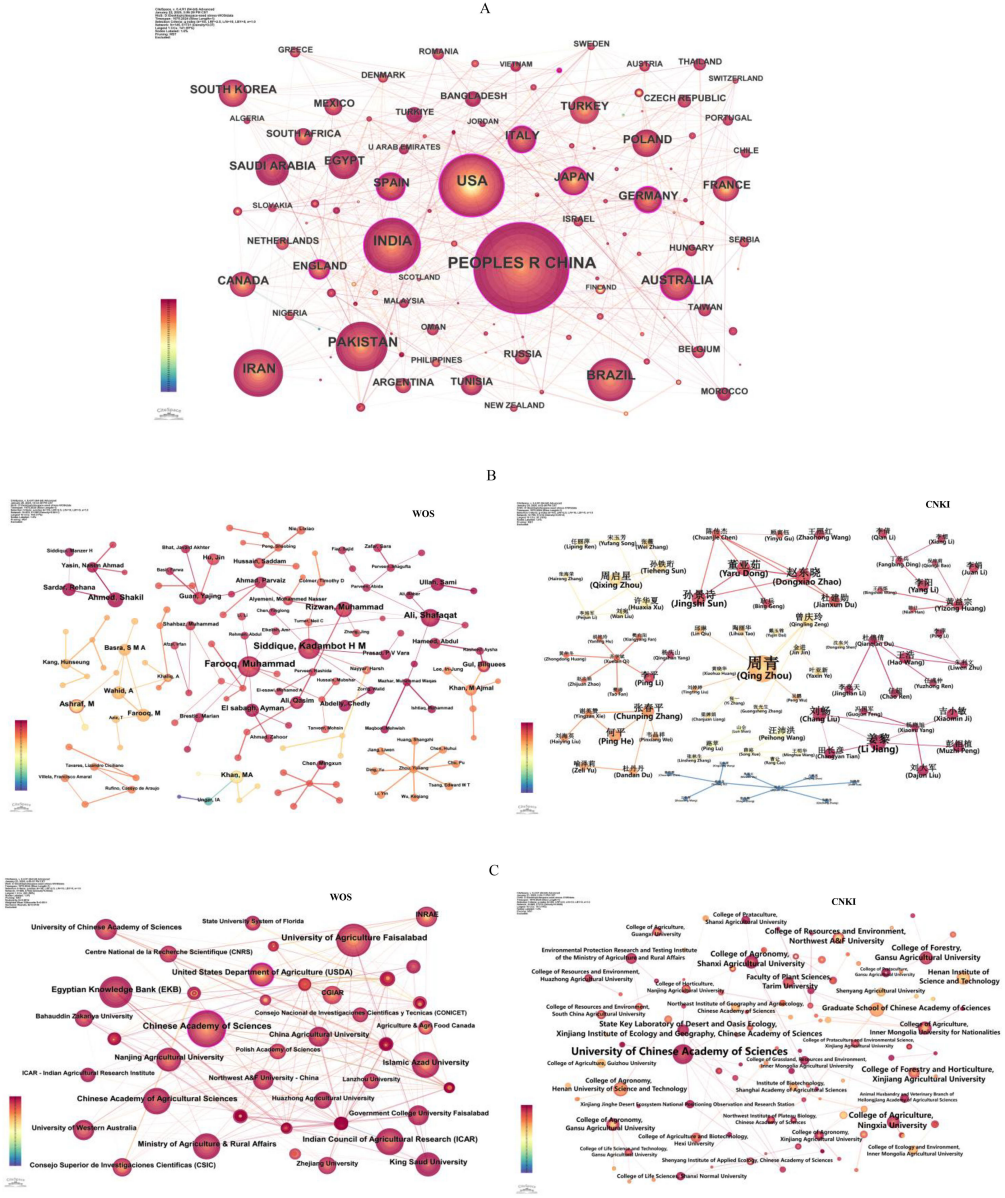
Multi-level cooperation network visualization in “seed-stress” research (1975–2024): Countries **(A)**, authors **(B)**, and institutions **(C)**. Nodes represent countries, authors, or institutions, with size indicating their publication output (larger nodes = more publications). Connecting lines indicate collaborative relationships between the corresponding entities.

The advancement of research on “seed-stress” is distinct across various countries. Delving into the geographical spread of scholarly contributions and collaborative actions helps reveal the research trends in this particular field (Chen et al., 2023; Zhu et al., 2025). The WOS country cooperation network (**Figure 3**) included 145 nodes (countries), 731 connections, and a network density (D) of 0.07, which is the ratio of the actual number of edges to the maximum possible number of edges (Keith et al., 2024). The top 5 productive countries (**Table 1**) were the People’s Republic of China (2,048 articles, 29.30%), the USA (1,106 articles, 15.83%), India (867 articles, 12.41%), Pakistan (648 articles, 9.27%), and Iran (597 articles, 8.54%). China ranked first in the number of publications in this field, becoming one of the core participants in international “seed-stress” research. However, there was still room for improvement in the closeness of cooperation with other countries. The low network density indicated that overall international cooperation had not yet formed a tight interactive pattern.

**Table 1:** Ranking of the top 10 most productive countries in terms of research output, based on the WOS database.

Rank	Countries	Publication number	Percentage (%)	First published year
1	PEOPLES R CHINA	2048	29.30	1986
2	USA	1106	15.83	1975
3	INDIA	867	12.41	1975
4	PAKISTAN	648	9.27	1998
5	IRAN	597	8.54	1977
6	BRAZIL	549	7.86	1992
7	AUSTRALIA	356	5.09	1975
8	JAPAN	284	4.06	1982
9	CANADA	269	3.85	1975
10	SAUDI ARABIA	265	3.79	1997

WOS denotes the Web of Science database.

To further elaborate on the international collaboration patterns, three distinct characteristics are identified based on the network structure and publication data:

First, a clear core-periphery structure dominates the collaboration network. The top 5 productive countries account for 75.35% of the total publications among the WOS top 10 countries, forming the core nodes. Peripheral nodes are mainly distributed in Southeast Asia, Africa, and South America, with relatively low publication output and passive collaboration status.

Second, the network exhibits low connectivity. This low density indicates that international collaboration is predominantly “bilateral shallow cooperation” rather than “multilateral in-depth collaboration”. For example, China’s cooperation with the USA and India primarily focuses on specific topics such as salt stress and heavy metal tolerance, without forming large-scale cross-regional research alliances.

Third, there is a regional agglomeration tendency in collaboration. South Asian countries (India, Pakistan) focus on drought and salt stress research in staple crops; Middle Eastern countries (Iran, Saudi Arabia) collaborate around high-temperature stress adaptation; and Sino-US-European collaborations center on molecular mechanisms (e.g., hormone regulation, antioxidant systems).

#### 3.2.2 Author cooperation networks

For the WOS Database, the author cooperation network (**Figure 3**) contained 833 nodes (representing authors), 385 connections, and a network density (D) of 0.0011. Within the author cooperation network, the circular elements (nodes) signify entities like authors or researchers involved in the network (Hou et al., 2024; Wu et al., 2024a). Among the top 10 authors in terms of publication volume (**Table 2**), Farooq, Muhammad (Farooq M.) ranked first with 33 literatures, focusing on the mechanism of stress responses during seed germination; Siddique, Kadambot H M (Siddique K.H.M.) ranked second with 31 literatures, concentrating on the physiological regulation of crop seeds under drought and high-temperature stress. Overall, cooperative relationships among international authors were relatively loose, with most authors conducting independent research or working in small teams, and large-scale cross-team collaboration was lacking.

**Table 2:** Top 10 most productive authors based on the WOS and CNKI databases over the period 1975-2024.

WOS				CNKI			
Rank	Author	Quantity	Year	Rank	Author	Quantity	Year
1	Farooq M.	33	2013	1	Zhou Q.	45	2000
2	Siddique K.H.M.	31	2017	2	Jiang L.	14	2021
3	Ashraf M.	26	2006	3	Liu C.	14	2020
4	Ali S.	23	2017	4	Wang L.X.	13	2007
5	Chauhan B.S.	20	2006	5	Du L.F.	13	2007
6	Ahmed S.	17	2009	6	He J.Y.	12	2008
7	Rizwan M.	17	2017	7	Ren Y.F.	12	2008
8	Ashraf M.	15	1993	8	Shao Y.	12	2005
9	Wang L.	15	2021	9	Zhao D.X.	12	2017
10	El sabagh A.	13	2020	10	Li X.C.	12	2005

WOS denotes the Web of Science database; CNKI denotes the China National Knowledge Infrastructure database.

For the CNKI Database, the author cooperation network included 799 nodes, 421 connections, and a network density (D) of 0.0013. Zhou, Qing (Zhou Q.) became the author with the highest number of publications in this field in China, with 45 literatures, focusing on low-temperature stress responses of wheat and rice seeds; Jiang, Li (Jiang L.) ranked second with 14 literatures, mainly focusing on the evaluation of salt stress tolerance during seed germination. Similar to the international network, domestic author cooperation also showed a “decentralized” characteristic, where only a few core authors formed stable cooperative teams, and the frequency of cooperation among most authors was low.

#### 3.2.3 Institutions cooperation networks

Investigating the institutions and collaborative efforts behind publications in this field can yield an accurate grasp of the distribution of research activities (Zhang, 2019). WOS database: The institution network (**Figure 3**) had 499 nodes, 643 connections, and D = 0.0052. The top 5 productive institutions were the Chinese Academy of Sciences (331 articles), University of Agriculture Faisalabad (255 articles), Egyptian Knowledge Bank (234 articles), Indian Council of Agricultural Research (177 articles), and United States Department of Agriculture (174 articles) (**Table 3**). CNKI database: The network (**Figure 2**) included 985 nodes, 272 connections, and D = 0.0006. The top 5 institutions were the University of Chinese Academy of Sciences (54 articles), Key Laboratory of Industrial Biotechnology (Jiangnan University, 32 articles), College of Agriculture (Inner Mongolia University for Nationalities, 31 articles), Graduate School of Chinese Academy of Sciences (27 articles), and College of Agronomy (Henan University of Science and Technology, 27 articles).

**Table 3:** Top 10 most productive institutions based on the WOS and CNKI databases over the period 1975-2024.

Database	Rank	Research institute	Count	Year
WOS	1	Chinese Academy of Sciences	331	1986
	2	University of Agriculture Faisalabad	255	2003
	3	Egyptian Knowledge Bank	234	1975
	4	Indian Council of Agricultural Research	177	1982
	5	United States Department of Agriculture	174	1981
	6	Chinese Academy of Agricultural Sciences	160	2008
	7	Islamic Azad University	158	2009
	8	Ministry of Agriculture & Rural Affairs	155	2009
	9	King Saud University	146	2010
	10	Nanjing Agricultural University	129	2008
CNKI	1	University of Chinese Academy of Sciences	54	2015
	2	Jiangnan University	32	2004
	3	Inner Mongolia University for Nationalities	31	2002
	4	Graduate School of Chinese Academy of Sciences	27	2005
	5	Henan University of Science and Technology	27	2006
	6	Shanxi Agricultural University	26	2011
	7	College of Forestry, Gansu Agricultural University	25	2011
	8	College of Agronomy, Gansu Agricultural University	24	2006
	9	Shangluo University	23	2016
	10	Henan Institute of Science and Technology	22	2005

WOS denotes the Web of Science database; CNKI denotes the China National Knowledge Infrastructure database.

### 3.3 Visual exploration of the intellectual base on “seed-stress” research

The intellectual base, comprising a thorough compilation of prior references in a specific field, primarily centers on examining the most frequently cited topics, their interconnections, and the relationships among these core subjects (Liu et al., 2024c). The top 10 most highly cited articles in WOS (**Table 4**) formed the core intellectual base of the field. The most highly cited was “Mechanisms of salinity tolerance”, authored by Munns R. and Tester M., with 8918 citations. This review comprehensively examined the mechanisms of salinity tolerance in plants at the cellular, organ, and whole-plant levels. Munns R. and Tester M. investigated the mechanisms of salinity tolerance from both physiological and molecular perspectives, clarifying that plant growth responds to salinity stress in two distinct phases: a rapid osmotic phase and a slower ionic phase. It also delved into the role of the HKT gene family in Na^+^ exclusion and its significance for plant salinity tolerance. However, the molecular mechanisms underlying whole-plant osmotic stress tolerance and overall regulation of Na^+^ accumulation remained poorly understood. Furthermore, the article explored the potential of molecular genetics and functional genomics to integrate molecular and physiological knowledge. These technological approaches were seen as holding promise to enhance our understanding of the molecular basis of plant salinity tolerance and to provide new strategies to improve salinity tolerance in plants. The second most highly cited article was “Reactive oxygen species and antioxidant defense in plants under abiotic Stress: Revisiting the crucial role of a universal defense regulator”, authored by Hasanuzzaman M. et al., with 1484 citations. This review explored the relationship between abiotic stress and reactive oxygen species (ROS) in plants, highlighting how adverse environmental conditions disrupted the balance between ROS production and antioxidant defenses, leading to oxidative stress. It underscored the critical role of both enzymatic and non-enzymatic antioxidant systems in detoxifying ROS and protecting plant cells. Additionally, the review examined the molecular crosstalk between ROS and other signaling molecules, such as reactive nitrogen species, sulfur, and carbonyl species, which were vital for plant stress responses and adaptation. The study also discussed the molecular mechanisms and regulatory strategies of ROS-mediated antioxidant defenses in enhancing plant tolerance to abiotic stress, pointing out the need for further research to fully understand these processes and develop stress-resistant crops. The third most highly cited article was “Zinc and iron oxide nanoparticles improved the plant growth and reduced the oxidative stress and cadmium concentration in wheat”, authored by Rizwan M. et al., with 555 citations. The study investigated the effects of zinc and iron oxide nanoparticles on wheat growth and their capacity to mitigate oxidative stress and cadmium accumulation. It was found that the application of these nanoparticles significantly enhanced plant biomass and photosynthetic efficiency. Additionally, they reduced levels of oxidative stress markers, such as malondialdehyde, and decreased cadmium concentrations in wheat plants. The nanoparticles likely acted by boosting the plants’ antioxidant defenses and regulating nutrient uptake. Among these 10 highly cited literatures, 8 were review articles, covering core directions such as salt stress, oxidative stress, and heavy metal stress. This indicated that review articles played a key role in integrating fragmented research results and guiding the research direction of the field.

**Table 4:** Top 10 most highly cited articles based on the WOS database.

Title	Author	Year	Journal	Article types	Institutions	Journal impact factor (5 years)	Citation
Mechanisms of salinity tolerance	Munns R. and Tester M.	2008	Annual Review of Plant Biology	Review;Book Chapter	CSIRO Plant Industry, Australia	28.4	8918
Reactive oxygen species and antioxidant defense in plants under abiotic Stress: Revisiting the crucial role of a universal defense regulator	Hasanuzzaman M., et al.	2020	Antioxidants	Review	Sher-e-Bangla Agricultural University	6.7	1484
Zinc and iron oxide nanoparticles improved the plant growth and reduced the oxidative stress and cadmium concentration in wheat	Rizwan M., et al.	2019	Chemosphere	Article	Government College University, Pakistan	7.7	555
Seed priming: state of the art and new perspectives	Paparella S., et al.	2015	Plant Cell Reports	Review	University of Pavia, Italy	5.3	497
Seed priming for abiotic stress tolerance: an overview.	Jisha K. C., et al.	2013	Acta Physiologiae Plantarum	Review	University of Calicut	2.8	418
Seed priming to alleviate salinity stress in germinating seeds	Ibrahim E. A.	2016	Journal of plant physiology	Review	Institute of Horticultural Research, Egypt	4.1	411
The signaling role of ROS in the regulation of seed germination and dormancy	Bailly C.	2019	Biochemical journal	Review	Sorbonne Université	3.7	202
Seed priming: a feasible strategy to enhance drought tolerance in crop plants	Marthandan V., et al.	2020	International journal of molecular sciences	Review	Tamil Nadu Agricultural University	5.6	161
Seed priming in field crops: potential benefits, adoption and challenges	Farooq M., et al.	2019	Crop and Pasture Science	Review	Sultan Qaboos University	2.1	148
APETALA 2-domain-containing transcription factors: focusing on abscisic acid and gibberellins antagonism	Shu K., et al.	2018	New Phytologist	Article	Sichuan Agricultural University, Institute of Ecological Agriculture	10.2	82

The intellectual base of “seed-stress” research is not only reflected in core highly cited articles but also in authoritative journals that serve as key academic communication platforms. **Table 5** presents the Top 10 most highly cited journals in this field based on the WOS database, including their ranking, number of published articles (Count), and 5-year impact factor. As shown in **Table 5**, the top three journals with the highest publication volume are Plant Physiology (4640 articles), Journal of Experimental Botany (3875 articles), and Frontiers in Plant Science (2651 articles), accounting for 38.7% of the total publications of the Top 10 journals, indicating a high concentration of core research outcomes in prestigious journals. In terms of academic influence, the 5-year impact factors of these journals range from 4.3 to 10.3, with New Phytologist (IF = 10.3) being the most influential one, followed by Plant Physiology and Plant Cell and Environment (both IF = 7.7). All these journals focus on plant stress physiology, seed development, or environmental adaptation mechanisms, which are highly consistent with the core theme of this study. The distribution of these highly cited journals further verifies the academic focus and international influence of “seed-stress” research, complementing the analysis of core articles (**Table 4**) to comprehensively depict the intellectual base of the field.

**Table 5:** Top 10 most highly cited journals based on the WOS database.

Rank	Journal	Count	Journal impact factor (5 years)
1	Plant Physiology	4640	7.7
2	Journal of Experimental Botany	3875	6.8
3	Frontiers in Plant Science	2651	5.7
4	Physiologia Plantarum	2616	5.4
5	Plant Physiology and Biochemistry	2427	6.4
6	Plant Science	2408	5.1
7	New Phytologist	2407	10.3
8	Plant Cell and Environment	2352	7.7
9	Journal of Plant Physiology	2301	4.3
10	Environmental and Experimental Botany	2250	5.4

### 3.4 Visual exploration of research hotspots on “seed-stress”

#### 3.4.1 Keyword co-occurrence network analysis

**Figure 4:**
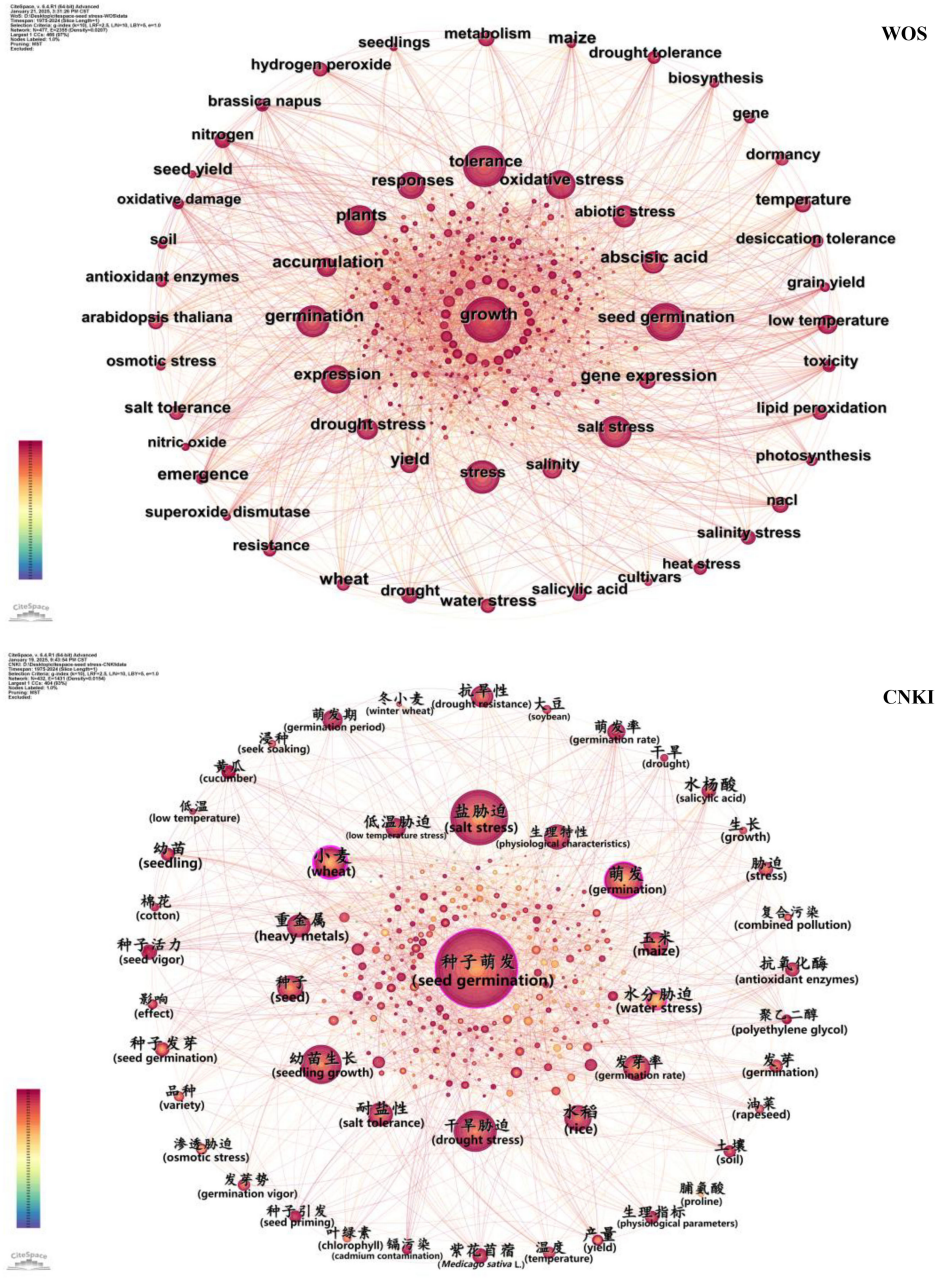
Visualization of keyword co-occurrence networks based on the WOS and CNKI databases. Nodes denote keywords, with size reflecting occurrence frequency (larger = more frequent).

Analyzing the frequency of keywords, which capture core elements such as aims, approaches, and viewpoints of a research article, is essential for identifying emerging topics and developmental trajectories within a specific field (Zhao et al., 2023). In keyword co-occurrence network analysis, the size of the circle in keyword co-occurrence network analysis serves as a visual indicator of the keyword’s importance or influence (Shen et al., 2024). WOS Database: The keyword co-occurrence network (**Figure 4**) included 477 nodes (representing keywords), 2,355 connections (representing co-occurrence relationships), and a network density (D) of 0.0207. The top 10 high-frequency keywords (**Table 6**) were “growth”, “tolerance”, “stress”, “seed germination”, “germination”, “plants”, “salt stress”, “oxidative stress”, “responses”, and “abscisic acid” in sequence. These keywords covered three dimensions: “stress type-physiological response-regulatory substance”, reflecting that international research focused on the growth and germination mechanisms of seeds under various stresses, as well as the regulatory role of hormones such as abscisic acid. CNKI Database: The keyword co-occurrence network contained 432 nodes, 1,431 connections, and a network density (D) of 0.0154. The core high-frequency keywords (**Table 6**) were “seed germination” (种子萌发), “salt stress” (盐胁迫), “seedling growth” (幼苗生长), “drought stress” (干旱胁迫), “germination” (萌发), “wheat” (小麦), “rice” (水稻), “maize” (玉米), “germination rate” (发芽率), and “salt tolerance” (耐盐性). Compared with international research, domestic keywords focused more on “specific crops-stress response indicators”, such as staple crops like wheat and rice, and indicators directly applicable to agricultural production such as germination rate, reflecting a strong application-oriented characteristic.

**Table 6:** Top 10 high-frequency keywords extracted from the WOS and CNKI databases.

Database	Rank	Keyword	Count	Year
WOS	1	growth	1857	1991
	2	tolerance	1387	1994
	3	stress	1182	1990
	4	seed germination	1178	1992
	5	germination	1061	1990
	6	plants	911	1991
	7	salt stress	864	1994
	8	oxidative stress	844	2003
	9	responses	780	1991
	10	abscisic acid	749	1991
CNKI	1	seed germination	1750	1998
	2	salt stress	994	1992
	3	seedling growth	590	1991
	4	drought stress	506	1992
	5	germination	489	1988
	6	wheat	441	1989
	7	rice	379	1998
	8	maize	350	1990
	9	germination rate	322	1994
	10	salt tolerance	297	1992

WOS denotes the Web of Science database; CNKI denotes the China National Knowledge Infrastructure database.

#### 3.4.2 Keyword clustering analysis

**Figure 5:**
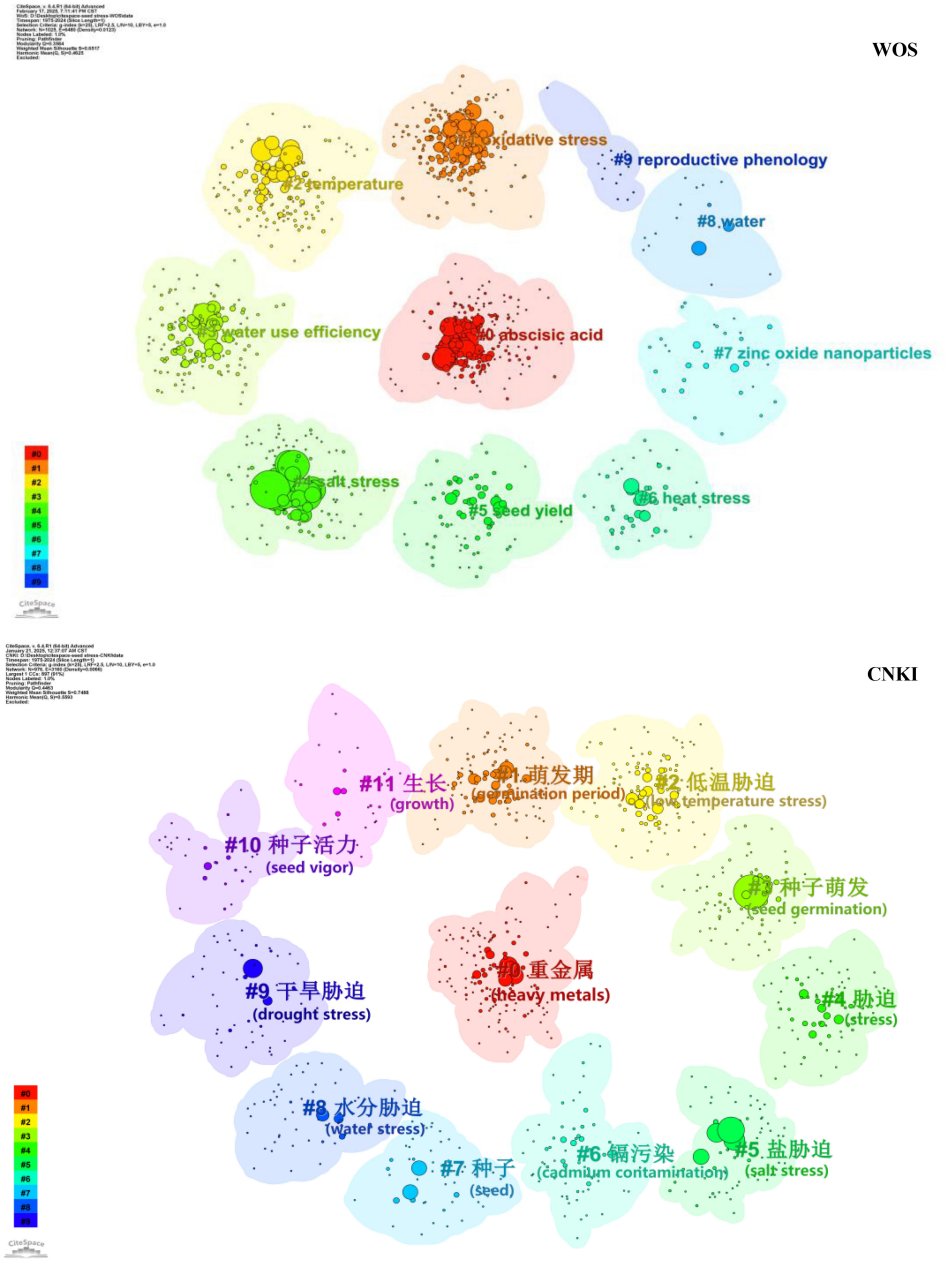
Visualization of keyword clustering based on the WOS and CNKI databases. Note: Nodes = keywords (size reflects occurrence frequency); different colors distinguish clusters (WOS: #0–#9; CNKI: #0–#11). All clusters have Silhouette Value (S Value) > 0.5 (range: 0.532–0.997 for WOS; 0.654–0.861 for CNKI), indicating reliable clustering results.

CiteSpace software was used for keyword clustering (Shen et al., 2024), and two indicators (Liu et al., 2025c)—modularity Q-value (measuring the significance of cluster structure, Q > 0.3 indicating significance) and silhouette coefficient S-value (measuring cluster clarity, S > 0.7 indicating excellence)—were used to evaluate the clustering effect:

For the WOS Database, the clustering results (**Figure 5**) had a Q-value of 0.3584 and an S-value of 0.6517, forming 10 clusters in total (**Table 7**). The three clusters with the largest number of nodes were #0 (abscisic acid, 206 nodes), #1 (oxidative stress, 191 nodes), and #2 (temperature, 155 nodes). The core keywords of Cluster #0 included “abscisic acid”, “Arabidopsis”, and “seed development”, focusing on the regulation of abscisic acid on seed development and stress responses; Cluster #1 centered on “oxidative stress”, “reactive oxygen species”, and “antioxidant enzymes”, delving into the physiological defense mechanisms of seeds under oxidative stress; Cluster #2 focused on “temperature”, “water stress”, and “light”, studying the interactive effects of multiple environmental factors on seeds.

**Table 7:** Comparative analysis of keyword clustering based on the WOS and CNKI databases over the period 1975-2024.

Database	Label	Node	S Value	Mean (Year)	Keywords
WOS	#0	206	0.639	2004	abscisic acid (292.16, 1.0 × 10^−4^); arabidopsis (193.15, 1.0 × 10^−4^); arabidopsis thaliana (138.92, 1.0 × 10^−4^); seed development (119.98, 1.0 × 10^−4^); expression (109.64, 1.0 × 10^−4^)
	#1	191	0.532	2011	oxidative stress (256.9, 1.0 × 10^−4^); reactive oxygen species (164.11, 1.0 × 10^−4^); antioxidant enzymes (154.24, 1.0 × 10^−4^); lipid peroxidation (103.57, 1.0 × 10^−4^); antioxidants (75.55, 1.0 × 10^−4^)
	#2	155	0.626	2009	temperature (233.52, 1.0 × 10^−4^); water stress (148.61, 1.0 × 10^−4^); light (129.87, 1.0 × 10^−4^); ph (124.43, 1.0 × 10^−4^); osmotic potential (102.07, 1.0 × 10^−4^)
	#3	137	0.573	2005	water use efficiency (76.16, 1.0 × 10^−4^); seed quality (70.59, 1.0 × 10^−4^); yield (58.43, 1.0 × 10^−4^); grain yield (53.44, 1.0 × 10^−4^); irrigation (49.24, 1.0 × 10^−4^)
	#4	124	0.671	2001	salt stress (158.41, 1.0 × 10^−4^); salinity (126.26, 1.0 × 10^−4^); seed priming (118.56, 1.0 × 10^−4^); seedling growth (95.55, 1.0 × 10^−4^); salt tolerance (77.06, 1.0 × 10^−4^)
	#5	67	0.734	2011	seed yield (152.87, 1.0 × 10^−4^); fatty acids (81.74, 1.0 × 10^−4^); antioxidant activity (77.39, 1.0 × 10^−4^); oil content (76.27, 1.0 × 10^−4^); fatty acid (57.79, 1.0 × 10^−4^)
	#6	48	0.843	2008	heat stress (189.74, 1.0 × 10^−4^); high temperature (83.32, 1.0 × 10^−4^); gas exchange (56.95, 1.0 × 10^−4^); pollen (53.65, 1.0 × 10^−4^); high temperature stress (50.47, 1.0 × 10^−4^)
	#7	31	0.886	2015	zinc oxide nanoparticles (37.94, 1.0 × 10^−4^); antioxidant system (32.22, 1.0 × 10^−4^); nanotechnology (30.34, 1.0 × 10^−4^); nanoparticles (23.72,1.0 × 10^−4^); nanomaterials (22.75, 1.0 × 10^−4^)
	#8	16	0.922	2001	water (32.94, 1.0 × 10^−4^); seed germination (16.67, 1.0 × 10^−4^); hydroponics (15.22, 1.0 × 10^−4^); cold stratification (15.22, 1.0 × 10^−4^); roots (15.22, 1.0 × 10^−4^)
	#9	16	0.997	1990	reproductive phenology (14.32, 1.0 × 10^−4^); supplemental mass pollination (14.32, 0.001); aerodynamics (14.32, 1.0 × 10^−4^); spore dispersal (14.32, 1.0 × 10^−4^); overhead cooling (14.32, 1.0 × 10^−4^)
CNKI	#0	141	0.761	2008	heavy metals (287.14, 1.0 × 10^−4^); soil (248.11, 1.0 × 10^−4^); rice (193.62, 1.0 × 10^−4^); wheat (173.05, 1.0 × 10^−4^); seed germination (164.34, 1.0 × 10^−4^)
	#1	118	0.712	2009	germination period (313.11, 1.0 × 10^−4^); salt resistance (185.78, 1.0 × 10^−4^); drought resistance (160.01, 1.0 × 10^−4^); membership functions (145.11, 1.0 × 10^−4^); germination Index (134.24, 1.0 × 10^−4^)
	#2	117	0.654	2010	low temperature stress (124.87, 1.0 × 10^−4^); physiological characteristics (121.03, 1.0 × 10^−4^); salicylic acid (118.11, 1.0 × 10^−4^); seedlings (69.16, 1.0 × 10^−4^); germination characteristics (68.65,1.0 × 10^−4^)
	#3	87	0.712	2006	seed germination (405.28, 1.0 × 10^−4^); cadmium stress (146.45, 1.0 × 10^−4^); cucumber (125.67, 1.0 × 10^−4^); soybean (77.31, 1.0 × 10^−4^); lead stress (62.03, 1.0 × 10^−4^)
	#4	74	0.739	2008	stress (98.81, 1.0 × 10^−4^); drought (86.37,1.0 × 10^−4^); temperature (79.87, 1.0 × 10^−4^); osmotic stress (66.39, 1.0 × 10^−4^); Medicago sativa L. (62.16, 1.0 × 10^−4^)
	#5	72	0.784	2004	salt stress (486.66, 1.0 × 10^−4^); seedling growth (298.73, 1.0 × 10^−4^); germination (285.74, 1.0 × 10^−4^); maize (160.87, 1.0 × 10^−4^); barley (27.44, 1.0 × 10^−4^)
	#6	65	0.788	2010	cadmium contamination (113.29, 1.0 × 10^−4^); phytoremediation (95.19, 1.0 × 10^−4^); passivators (82.45, 1.0 × 10^−4^); safe utilization (65.58, 1.0 × 10^−4^); rapeseed (49.43, 1.0 × 10^−4^)
	#7	58	0.752	2010	seed (312.04, 1.0 × 10^−4^); germination rate (184.65, 1.0 × 10^−4^); tissue culture (114.43, 1.0 × 10^−4^); seed germination (76.14, 1.0 × 10^−4^); salinity stress (55.97, 1.0 × 10^−4^)
	#8	53	0.82	2002	water stress (209.15, 1.0 × 10^−4^); physiological indicators (76.06, 1.0 × 10^−4^); growth indicators (32.06, 1.0 × 10^−4^); malondialdehyde (29.19, 1.0 × 10^−4^); abscisic acid (29.02, 1.0 × 10^−4^)
	#9	50	0.786	2009	drought stress (325.79, 1.0 × 10^−4^); germination (88.78, 1.0 × 10^−4^); electric field (40.14, 1.0 × 10^−4^); salicornia (28.66, 1.0 × 10^−4^); enzyme activity (25.97, 1.0 × 10^−4^)
	#10	32	0.849	2005	seed vigor (119.18, 1.0 × 10^−4^); cause (30.54, 1.0 × 10^−4^); tobacco (30.54, 1.0 × 10^−4^); peas (28.79, 1.0 × 10^−4^); plant hormones (20.12, 1.0 × 10^−4^)
	#11	30	0.861	2012	growth (99.61, 1.0 × 10^−4^); physiology and biochemistry (76.33, 1.0 × 10^−4^); effect (48.45, 1.0 × 10^−4^); plant (32.34, 1.0 × 10^−4^); osmoregulation (31.37, 1.0 × 10^−4^)

WOS denotes the Web of Science database; CNKI denotes the China National Knowledge Infrastructure database.

For the CNKI Database, the clustering results had a Q-value of 0.4463 and an S-value of 0.7488, forming 12 clusters in total. The key clusters included #0 (heavy metals, 141 nodes), #1 (germination period, 118 nodes), and #2 (low-temperature stress, 117 nodes). The core keywords of Cluster #0 were “heavy metals” (重金属), “soil” (土壤), “rice” (水稻), and “wheat” (小麦), addressing the issue of heavy metal pollution in domestic farmland and studying its impact on crop seed germination; Cluster #1 focused on “germination period” (萌发期), “salt tolerance” (耐盐性), and “drought tolerance” (抗旱性), concentrating on the evaluation of stress tolerance during seed germination; Cluster #2 centered on “low-temperature stress” (低温胁迫), “salicylic acid” (水杨酸), and “seedlings” (幼苗), exploring the impact of low temperature on crop seed germination and seedling growth, as well as the mitigating effect of exogenous substances such as salicylic acid.

### 3.5 Visual exploration of dynamic frontiers

#### 3.5.1 Evolution trends

**Figure 6:**
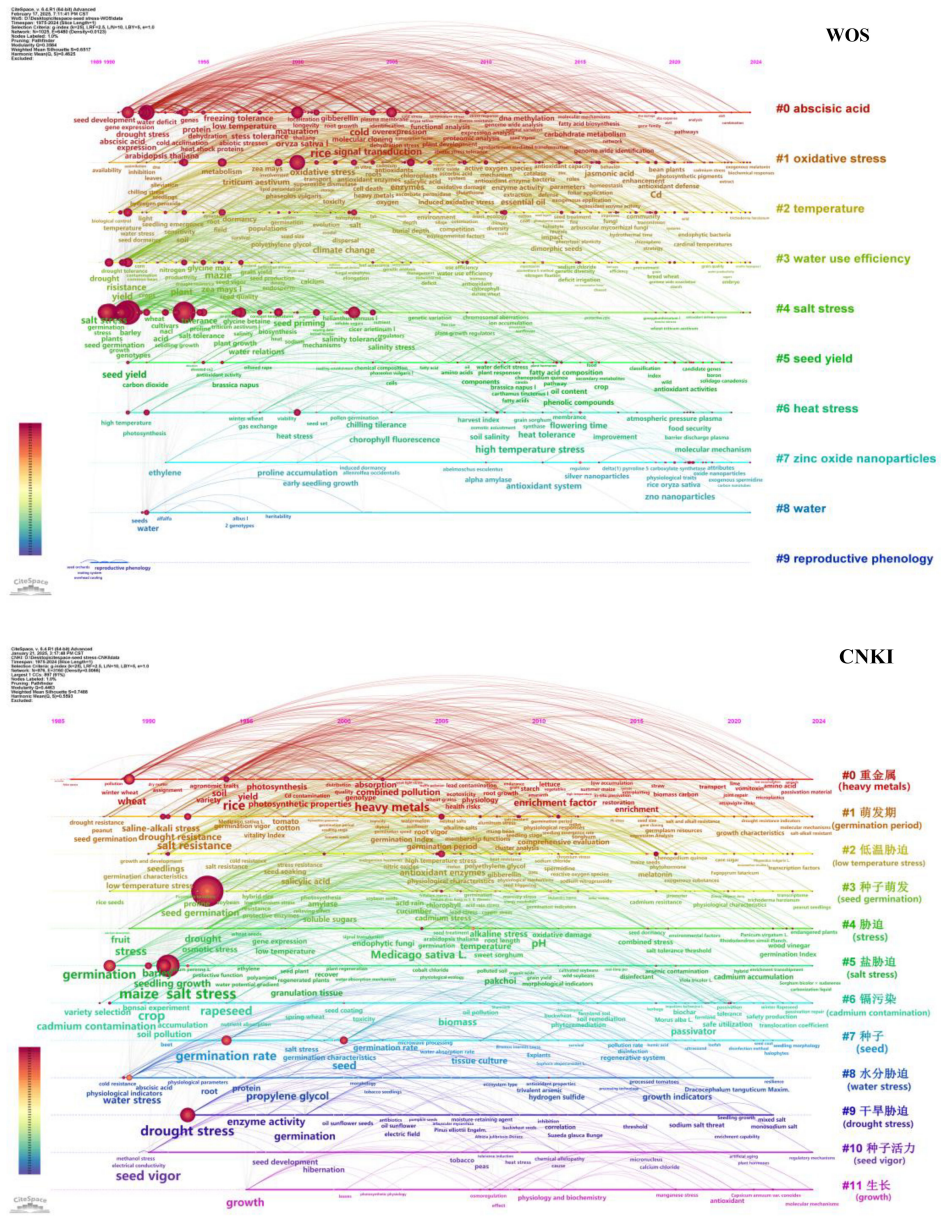
Timeline mapping of keyword clustering based on the WOS and CNKI databases. Note that clusters are arranged vertically, time flows horizontally from left to right, and keyword activity bursts within each cluster are marked along the timelines.

Utilizing CiteSpace method, a timeline map of keyword clustering was constructed, elucidating the chronological span and developmental trajectories of nascent research topics within the field of “seed-stress” (Wang et al., 2025b). Combined with the keyword clustering timeline (**Figure 6**) and quantitative indicators (**Table 7**), the research evolution of the two databases showed significant differences:

##### 3.5.1.1 The WOS database

High-stability clusters: #9 (reproductive phenology, S = 0.997, mean year = 1990) and #7 (zinc oxide nanoparticles, S = 0.886, mean year = 2015). Cluster #9 was a fundamental direction of early research, with keywords such as “reproductive phenology” and “spore dispersal” having a frequency of 14.32. Although the research intensity was low, the S-value close to 1 indicated its fundamental position in the field of plant reproductive ecology; Cluster #7 was an emerging frontier direction, with keywords such as “zinc oxide nanoparticles” (frequency: 37.94) and “antioxidant system” (frequency: 32.22). The high S-value reflected a high degree of research focus on “nano-agriculture” in seed stress mitigation. Evolution of core clusters: Cluster #0 (abscisic acid, 206 nodes) had a mean year of 2004, and the frequency of the keyword “abscisic acid” was 292.16 (the highest among all clusters). This direction had been continuously active since 2000 and was a core field in seed development and hormone regulation; Cluster #1 (oxidative stress, 191 nodes) had a mean year of 2011, and the keyword density increased significantly after 2010, reflecting that research on oxidative stress mechanisms entered an in-depth development stage after 2010; Cluster #2 (temperature, 155 nodes) had a mean year of 2009, integrating multiple environmental factors such as temperature, water, and light, and became a key platform for cross-stress research.

##### 3.5.1.2 The CNKI database

High-stability clusters: #5 (salt stress, S = 0.784), #9 (drought stress, S = 0.786), and #6 (cadmium contamination, S = 0.788). These three clusters corresponded to typical domestic environmental issues—saline-alkali land in northern China, arid areas in western China, and cadmium-contaminated farmland in southern China. The frequency of the keyword “salt stress” was 486.66, and that of “drought stress” was 325.79. The high S-values and frequencies indicated that these directions were long-term key research fields in China. Evolution of core clusters: Cluster #0 (heavy metals, 141 nodes) had a mean year of 2008, the frequency of the keyword “heavy metals” was 287.14, and the frequencies of “rice” and “wheat” both exceeded 170, focusing on the impact of domestic heavy metal pollution on staple crop seeds; Cluster #1 (germination period, 118 nodes) had a mean year of 2009, and the frequency of the keyword “germination period” was 313.11 (the highest among all clusters). Aiming at the problem of low seedling emergence rate caused by spring drought and saline-alkali land in northern China, it studied stress tolerance during the germination period; Cluster #2 (low-temperature stress, 117 nodes) had a mean year of 2010, the frequency of the keyword “low-temperature stress” was 124.87, and that of “salicylic acid” was 118.11. Focusing on the harm of “late spring cold” to early rice in southern China and wheat in northern China, it explored low-cost exogenous regulatory technologies.

#### 3.5.2 Research frontiers

CiteSpace’s burst detection functionality is employed to measure the prevalence and temporal emergence of keywords within academic literature, thereby delineating the dynamic frontiers and potential trajectory of research on “seed-stress” within the field (Han et al., 2024). Keywords that surface and persist for a minimum of two years typically signify an emerging trend, which can aid in forecasting future research hotspots and developmental pathways (Zhang et al., 2024). Using the keyword burst function of CiteSpace (identifying emerging research directions, with a burst duration ≥ 2 years considered an effective frontier), the research frontiers of the two databases showed significant differences (**Table 8**).

**Table 8:** Comparative analysis of outburst keywords based on the WOS and CNKI databases.

Top 20 Keywords with the Strongest Citation Bursts (WOS)					
Keywords	Year	Strength	Begin	End	1975 - 2024
stress	1990	26.34	1990	2003	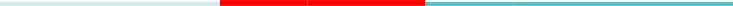
water stress	1991	56.42	1991	2009	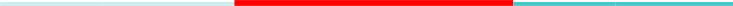
moisture stress	1991	16.06	1991	2010	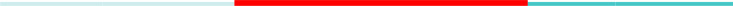
plants	1991	16.06	1992	2002	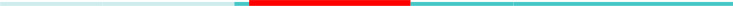
barley	1992	15	1992	2012	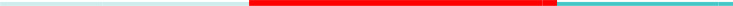
tomato	1994	12.45	1994	2012	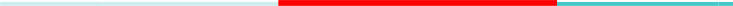
desiccation tolerance	1994	11.34	1994	2007	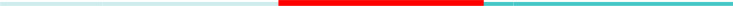
germination	1990	17.02	1995	2006	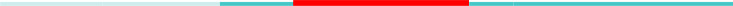
maize	1995	12.41	1995	2006	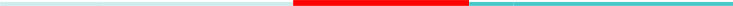
temperature	1991	31.57	1997	2010	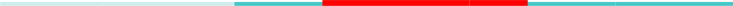
emergence	1994	20.07	1998	2009	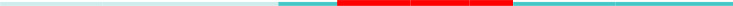
salinity	1997	12.41	2001	2007	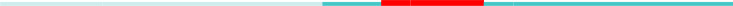
thaliana	2001	11.59	2001	2018	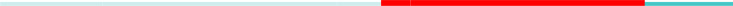
nacl	1997	17.49	2002	2013	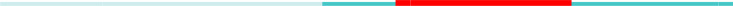
seed yield	1995	11.59	2005	2013	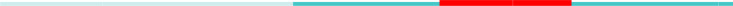
signal transduction	2008	17.83	2008	2016	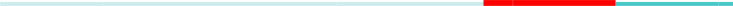
salt	2003	12.74	2008	2014	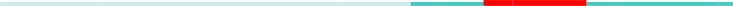
foliar application	2020	11.66	2021	2024	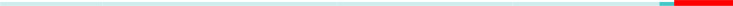
antioxidant defense	2021	15.3	2022	2024	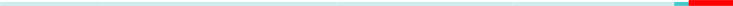
nanoparticles	2022	11.18	2022	2024	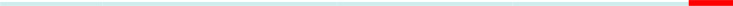

Top 20 Keywords with the Strongest Citation Bursts (CNKI)					
Water stress	1989	37.54	1989	2009	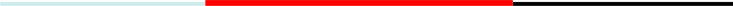
proline	1993	9.68	1993	2011	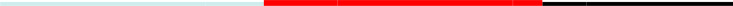
drought resistance	1991	9.41	1996	2005	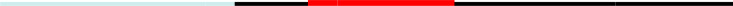
salinity stress	1998	8	1998	2007	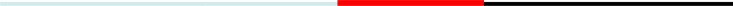
amylase	1999	8.19	1999	2009	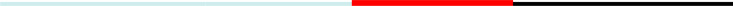
acid rain	2004	12	2004	2010	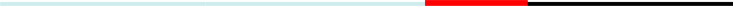
cucumber	2004	7.53	2004	2009	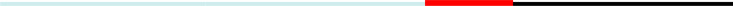
seed germination	1988	8.31	2005	2008	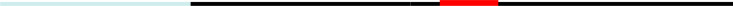
acid rain stress	2007	9.56	2007	2011	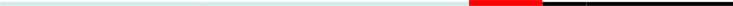
peas	2007	7.79	2007	2010	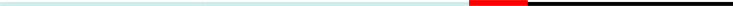
yield	1995	12.72	2008	2013	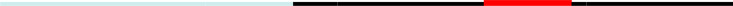
heavy metals	2002	9.76	2013	2015	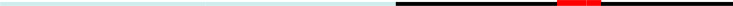
phytoremediation	2012	7.26	2018	2019	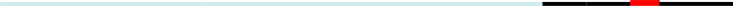
cadmium contamination	1988	7.86	2019	2024	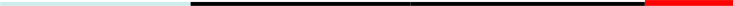
germination period	2005	12.16	2020	2024	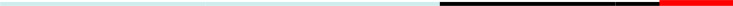
membership functions	2007	10.08	2021	2024	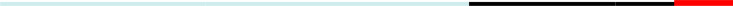
cluster analysis	2009	7.41	2021	2024	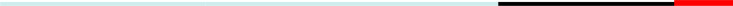
melatonin	2016	17.66	2022	2024	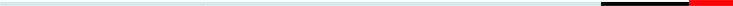
comprehensive evaluation	2011	12.36	2022	2024	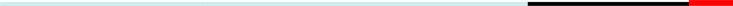
seed induction	2009	7.87	2022	2024	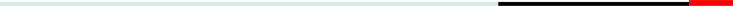

WOS denotes the Web of Science database; CNKI denotes the China National Knowledge Infrastructure database.

For the WOS Database, focusing on international cutting-edge technologies and basic mechanisms, high-burst-intensity keywords included “water stress” (strength: 56.42), “temperature” (31.57), “signal transduction” (17.83), and “nanoparticles” (11.18). Emerging frontiers after 2020 included “foliar application”, “antioxidant defense”, and “nanoparticles”, focusing on the combination of technology application and molecular mechanisms. CNKI Database: Guided by domestic practical problems and practical technologies, high-burst-intensity keywords included “water stress” (strength: 37.54), “acid rain” (12.00), “cadmium contamination” (7.86), and “membership functions” (10.08). Emerging frontiers after 2020 included “cadmium contamination control”, “germination stage regulation”, and “melatonin application”, focusing on pollution control and the practical application of seed regulatory technologies. Overall, WOS serves global scientific frontier breakthroughs, while CNKI supports domestic agricultural production and environmental governance.

## 4 Discussion

### 4.1 Driving factors for temporal dynamics of publications

The three-stage publication trend in “seed-stress” research is essentially the result of the combined effects of “environmental demand, technological advancement, and policy orientation”. In Stage I (1975–2003), international research took the lead in exploring seed stress responses, relying on advanced physiological observation technologies (e.g., gas chromatography for hormone content determination) and long-standing foundations in plant physiology. In contrast, domestic research started relatively late due to constraints in technical conditions and funding investment (Liu et al., 2024b). During Stage II (2004–2019), the rapid growth in the number of domestic literatures was closely linked to national policy support. Since 2004, China has launched projects such as the “National Science and Technology Support Program” and “Agricultural Science and Technology Achievement Transformation Fund”, which focused on supporting research on stress-resistant crop breeding and cultivation technologies. In 2015, the National Plan for Sustainable Agricultural Development (2015–2030) explicitly proposed to “enhance crop stress resistance”, further promoting domestic “seed-stress” research (Bryan et al., 2018). Meanwhile, domestic research institutions gradually introduced molecular biology technologies (e.g., RT-PCR, proteomics), significantly improving the depth of research and narrowing the gap with international studies (Cai et al., 2025). Stage III (2020–2024) has entered a “steady breakthrough phase”. On one hand, the global scientific evaluation system has shifted from “quantity-oriented” to “quality-oriented”, with research focusing more on depth rather than breadth. On the other hand, extreme climate events (e.g., the 2022 global high temperatures, the 2023 drought in North China, China) have driven research to focus on specialized directions such as “interaction of multiple stresses” and “molecular regulatory mechanisms”. For instance, international research has focused on nanomaterials to alleviate combined stresses, while domestic research has focused on the synergistic effects of cadmium contamination and drought (Xu et al., 2022; Wu et al., 2024b).

### 4.2 Characteristics and issues of spatial cooperation networks

#### 4.2.1 Characteristics and limitations of international collaboration patterns

The international collaboration pattern in “seed-stress” research is shaped by two key driving factors. On one hand, differences in research priorities create collaboration barriers: China focuses on stress responses of staple crops (wheat, rice), while European and American countries prioritize model plants (*Arabidopsis thaliana*) and cash crops (maize, soybean), leading to limited overlapping research areas. On the other hand, language and cultural barriers hinder in-depth cooperation—some domestic research results are only published in Chinese journals, reducing accessibility for international peers and limiting the expansion of collaborative projects (Chen et al., 2023).

From the perspective of institutional collaboration, core institutions such as the Chinese Academy of Sciences (CAS), the United States Department of Agriculture (USDA), and the Indian Council of Agricultural Research (ICAR) mainly engage in “project-based short-term cooperation”. For example, CAS and USDA jointly published one paper on “seed priming technology for stress resistance” but did not extend the collaboration to molecular mechanism analysis or field verification. This “one-time cooperation” model fails to establish stable knowledge-sharing mechanisms, restricting the depth and sustainability of collaborative research.

Additionally, the “core-periphery” structure exacerbates collaboration imbalance. Core countries (China, USA, and India) occupy over 75% of research funds and advanced equipment resources, while peripheral countries (e.g., Pakistan, Kenya) are mostly in passive roles such as sample collection and basic data recording, lacking participation in core experimental design and data analysis. This imbalance makes it difficult to form systematic research conclusions covering different climate zones and crop types, such as the lack of a seed stress resistance evaluation system suitable for tropical regions due to insufficient participation of peripheral countries like Brazil and Indonesia.

#### 4.2.2 Fragmentation of domestic and international author-institution collaboration

The low-density characteristics of author and institutional cooperation networks (WOS author network D = 0.0011; CNKI institutional network D = 0.0006) further expose the fragmentation of research collaboration. From the author perspective, the cooperation of international core scholars shows a “small circle” feature: the Farooq, M. team has long focused on oxidative stress and germination mechanisms of soybean and wheat seeds. In 2017, they collaborated with Huth et al. to analyze the impact of lignin content in soybean seed coats on oxidative stress sensitivity (Huth et al., 2017), and in 2024, they jointly worked with Ji et al. to explore the relationship between oxidative stress and viability of *Paeonia lactiflora* seeds during cryopreservation (Ji et al., 2024). Their collaborators have always been limited to the field of plant physiology, and no interdisciplinary collaboration has been formed with scholars in fields such as nanomaterials and molecular genetics, making it difficult to break through the single-dimensional research framework (Zhang et al., 2020). Domestic core authors also have similar limitations: the Zhou, Q. team has focused on low-temperature stress responses of wheat and tobacco seeds. In 2015, they studied the impact of low temperature and long-term storage on tobacco seed germination (Lu, 2015), and in 2018, they analyzed the effect of low-temperature stress on seedling emergence of direct-seeded early rice (Duan et al., 2018). Although the research objects have expanded, the team has always focused on the single direction of “low-temperature stress” and lacks academic exchanges with teams studying cadmium pollution in rice or salt stress in maize, resulting in difficulties in integrating knowledge across crop types and stress categories.

From the institutional perspective, in the WOS database, although the Chinese Academy of Sciences (CAS), as an international core institution, has cooperation with the United States Department of Agriculture (USDA) and the Indian Council of Agricultural Research (ICAR), most of these collaborations are “project-based short-term cooperation”. For example, after jointly publishing one paper with the USDA on “seed priming technology to improve stress resistance”, there was no further expansion to molecular mechanism analysis or field verification; cooperation with ICAR also only stayed at the phenotypic observation of wheat under salt stress, without involving core scientific issues such as gene regulation and protein interaction (Keith et al., 2024). This “one-time cooperation” model makes it difficult to form a stable knowledge-sharing mechanism, leading to challenges in sustaining in-depth research. In the CNKI database, the “regional fragmentation” of institutional collaboration is more obvious: Inner Mongolia University for Nationalities focuses on low-temperature issues in northern China and conducted a study on the impact of low temperature on early rice seedling growth in 2018 (Duan et al., 2018); the Key Laboratory of Industrial Biotechnology of the Ministry of Education (Jiangnan University) focuses on microbial regulation of seed stress responses; Henan University of Science and Technology pays attention to salt and drought stress of crops in the Huang-Huai-Hai region. Affected by “localized research resource allocation” and “regional agricultural problem orientation”, it is difficult for institutions to form cross-regional research alliances, and there is even duplication of research content.

In addition, the “core-periphery” structure of the cooperation network exacerbates the imbalance of collaboration. Internationally, core countries/institutions such as the United States, China, and India occupy more than 75% of research funds and advanced equipment resources. Peripheral countries (such as some countries in Southeast Asia and Africa) are mostly in a “passive collaboration” position due to funding shortages and backward technology. For example, when Pakistani scholars participate in Chinese teams’ research on wheat salt stress, they only undertake field sample collection and basic data recording, while the core experimental design and data analysis are led by Chinese teams; the gene sequencing and transcriptome analysis of the 2022 paper “Maize Seed Response to High-Temperature Stress” published by Kenyan scholars in WOS relied on technical support from Cornell University (USA), making it difficult for local teams to participate in discussions on core scientific issues. Domestically, research resources are concentrated in CAS and 985/211 universities, while local colleges and universities mostly conduct research as “followers”. For example, a 2024 paper on “Cassia obtusifolia Seed Response to Salt Stress” (CNKI Cluster #5) published by a local agricultural university completely adopted the experimental method of Henan University of Science and Technology’s 2020 study, only changing the crop variety without proposing original research ideas. This structure causes core nodes to hold the right to speak in research, while peripheral nodes can only follow passively, making it difficult to form systematic research conclusions covering different climate zones and crop types. For example, global research on “high-temperature stress of tropical crop seeds” has not yet established a seed stress resistance evaluation system suitable for tropical regions due to the lack of in-depth participation of peripheral countries such as Brazil and Indonesia (Zhu et al., 2025).

### 4.3 Core role and limitations of the intellectual base

The 8 highly cited review papers in the WOS database (**Table 4**) constitute the knowledge hub of the “seed-stress” field, and their core value is reflected in three aspects: first, integrating fragmented research results. For example, Mechanisms of salinity tolerance published by Munns and Tester (2008) (cited 8,918 times) systematically sorted out the “two-phase response” mechanism of plants to salt stress, clarified the role of the HKT gene family in sodium ion exclusion, and provided a unified theoretical framework for subsequent studies on seed salt stress responses; second, guiding research directions. Reactive oxygen species and antioxidant defense in plants under abiotic stress by Hasanuzzaman et al. (2020) (cited 1,431 times) established the “stress-ROS-antioxidant system” research paradigm, promoting in-depth development of international research on oxidative stress mechanisms after 2010—for instance, Ji et al.’s (2024) study on oxidative stress of Paeonia lactiflora seeds was based on this paradigm; third, building technical bridges. Zinc and iron oxide nanoparticles improved the plant growth by Rizwan et al. (2019) (cited 555 times) confirmed the stress-alleviating effect of nanomaterials, providing empirical support for the integration of “nano-agriculture” with seed stress research.

However, the intellectual base also has obvious limitations: first, the low proportion of empirical studies (only 2 out of 10), reflecting the field’s over-reliance on theoretical integration and insufficient original experimental breakthroughs. For example, current research on “hormone interaction mechanisms under seed stress” mostly carries out verification experiments based on the theoretical framework of Munns and Tester (2008), lacking exploration of new regulatory factors (such as non-coding RNA); second, the single research perspective. Highly cited papers mostly focus on plant self-responses, ignoring the “seed-microbe-soil” interaction—for example, they do not involve the regulatory role of rhizosphere microbes in seed stress responses, making it difficult for research to reflect actual conditions in complex field environments (Zhao et al., 2023); third, insufficient geographical representation. 70% of highly cited papers come from European and American countries, and only 30% involve research from major agricultural countries such as China and India, making it difficult to cover agricultural production needs in different regions. For example, seed regulatory technologies for saline-alkali land and cadmium-contaminated farmland are rarely mentioned in highly cited papers.

### 4.4 Differences in research hotspots and integration paths

The difference between international and domestic research hotspots essentially reflects the divergence between “basic research orientation” and “application demand orientation”. Hotspots in the WOS database focus on “mechanism analysis”: Cluster #0 (abscisic acid) centers on hormone regulation of seed development and stress responses—for example, Li et al. (2016) studied the synergistic regulation of abscisic acid and gibberellin on *Suaeda salsa* seed germination; Cluster #1 (oxidative stress) explores the regulatory mechanisms of ROS production and antioxidant enzyme systems in depth—such as Huth et al. (2017) analyzing factors affecting oxidative stress sensitivity of soybean seeds; Cluster #2 (temperature) focuses on the interaction of multiple environmental factors (temperature, water, light)—for instance, Stefanello et al. (2017) explored the germination vitality of flaxseeds under combined stress. This distribution of hotspots stems from the “basic research priority” positioning of European and American countries, as well as their resource inclination towards model plants (*Arabidopsis thaliana*) and advanced technologies (such as gene editing).

Hotspots in the CNKI database are closely linked to “domestic agricultural issues”: Cluster #0 (heavy metals) targets cadmium-contaminated farmland in southern China, studying the heavy metal accumulation mechanism of rice and wheat seeds—for example, Ge et al. (2002) analyzed the impact of heavy metals on amylase activity in germinating rice seeds; Cluster #1 (germination period) focuses on the low seedling emergence rate caused by spring drought and saline-alkali land in northern China, conducting evaluations of stress tolerance during germination—such as Chai et al. (2008) studying the response of sweet sorghum to salt stress during germination; Cluster #2 (low-temperature stress) explores the harm of “late spring cold” to early rice and wheat and corresponding regulatory technologies—for instance, Duan et al. (2018) analyzed the impact of low temperature on early rice seedling growth. This distribution of hotspots is highly consistent with China’s policy orientation of “research serving production”. For example, the National Plan for Sustainable Agricultural Development (2015–2030) explicitly requires “solving prominent agricultural ecological and environmental problems”, which directly promotes research in directions such as heavy metals and salt stress (Wang et al., 2024; Song et al., 2025).

The integration of these two types of hotspots can be advanced from three aspects: first,“mechanism-application” transformation—applying international molecular mechanism research to domestic crop improvement. For example, based on the abscisic acid regulation mechanism of WOS Cluster #0, developing seed priming technologies for wheat and rice to alleviate the impact of low temperature and salt stress (Ashraf and Abushakra, 1978; Jisha et al., 2013; Zhang et al., 2025); second, “problem-theory” refinement—elevating domestic practical problems into basic research propositions. For example, exploring new heavy metal tolerance genes based on the seed response characteristics of cadmium-contaminated farmland to supplement the international theoretical system; third, “technology-region” adaptation—combining international advanced technologies (such as nanomaterials, gene editing) with domestic regional environments. For example, adjusting the application parameters of zinc oxide nanoparticle technology (WOS Cluster #7) for northern saline-alkali land and southern cadmium-contaminated farmland to improve technical applicability (Bryan et al., 2018; Wu et al., 2024b).

### 4.5 Evolution of dynamic frontiers and future research directions

The evolution of international and domestic research frontiers presents a dual-track feature of “technology-driven” and “problem-driven”. Frontiers in the WOS database have evolved from “basic physiology” to “technology application”: early (1990s) focusing on basic directions such as reproductive phenology (Cluster #9), mid-term (2010s) shifting to mechanism research such as oxidative stress and hormone regulation (Clusters #1, #0), and recent (2020s) centering on technologies such as nanomaterials (Cluster #7) and foliar application. For example, studies on zinc oxide nanoparticles alleviating salt and water stress from 2022 to 2024 (literatures in WOS Cluster #7) reflect the logic of “technological breakthroughs promoting frontier expansion” (Moreira et al., 2024; Naaz et al., 2025).

Frontiers in the CNKI database have evolved from “problem identification” to “solution development”: early (2000s) identifying domestic environmental issues such as salt stress and heavy metal pollution (Clusters #5, #0), mid-term (2010s) exploring technologies such as germination regulation and low-temperature defense (Clusters #1, #2), and recent (2020s) focusing on practical solutions such as cadmium pollution control and melatonin application (**Table 7**). For instance, a 2025 study conducted a comprehensive evaluation of salt tolerance in soybean germplasm resources during the germination stage. By measuring indicators such as germination energy, germination rate, and radicle length, three soybean germplasm accessions with excellent salt tolerance were screened out. Additionally, this study clarified the correlation between salt tolerance at the germination stage and salt stress response at the seedling stage of soybeans (Han et al., 2025; Ul Haq et al., 2025). Not only did this research align with the focus on “stress tolerance evaluation” in CNKI Cluster #1 (“germination period”), but it also advanced the applied research of Cluster #5 (“salt stress”). Addressing the low seedling emergence rate caused by salinization in major soybean-producing areas of China, it provided directly applicable germplasm resources and evaluation methods for salt-tolerant soybean breeding, embodying the logic of “demand orientation driving the deepening of research frontiers” (Han et al., 2025). Combining the evolutionary characteristics of these two types of frontiers, future research can focus on three directions: first, mechanisms and regulation of combined stresses—integrating international molecular mechanisms (such as ROS signaling, hormone interaction) with domestic crop research to establish a regulatory network for multi-stress responses, targeting combined stresses such as “high temperature-drought” and “salt-cadmium” under extreme climates (Bailly, 2019; Marthandan et al., 2020; Sun et al., 2025); second, development and application of green technologies—combining frontier technologies in WOS (such as nanomaterials, biostimulants like melatonin) with domestic farmland realities to develop low-cost and easy-to-operate seed treatment technologies, such as nanoparticle-coated seeds and melatonin-soaked seeds; third, cross-scale collaboration and data sharing—establishing a “global-regional” linked research network. For example, China can collaborate with Southeast Asian countries to conduct research on “high-temperature stress of tropical crop seeds”, while European and American countries can join forces with African teams to explore “stress-resistant breeding of seeds in arid areas”. At the same time, building an open seed stress database to integrate research data from WOS and CNKI, promoting collaborative development of the field.

## 5 Conclusions and outlook

In the face of intensifying environmental stress, investigating how plant seeds respond to such conditions is of paramount importance. This study leveraged CiteSpace v.6.4.R1 to conduct an in-depth analysis of 15627 pertinent scholarly records from the WOS and CNKI databases, covering the period from 1975 to 2024. Regarding temporal publication trends, research on “seed-stress” has traversed three distinct phases: the initial exploration phase (1975-2003), the rapid growth phase (2004-2019), and the steady breakthrough phase (2020-2024). Both international (WOS) and domestic (CNKI) research exhibit an upward trajectory in annual publication volume, with an average annual increase of 684 articles in the WOS database and 453 articles in the CNKI database since 2020. The cumulative publication volume in the WOS database (9042 articles) exceeds that in the CNKI database (6585 articles), indicative of a sustained global scientific focus on this domain. In terms of cooperation networks among countries, authors, and institutions, China leads in publication volume within the WOS database, playing a pivotal role in international collaboration. However, the cooperation networks of authors and institutions in both international and domestic research require enhancement. The network density of author cooperation is exceedingly low in both databases, suggesting limited and sporadic collaboration. Regarding institutions, while the WOS database exhibits a more interconnected network, the CNKI database encompasses a greater number of participating institutions with less interconnection. Analyses of the intellectual base, research hotspots, and dynamic frontiers reveal disparities between international and domestic research. The international intellectual base primarily comprises articles on salinity tolerance and oxidative stress, with most highly-cited articles being review articles. In keyword co-occurrence network analysis, the WOS database features a larger and more interconnected network, reflecting a diverse and integrated research landscape, whereas the CNKI database is more focused and fragmented. Keyword clustering analysis indicates that the CNKI database has a more robust community structure, with more distinct and well-defined keyword clusters. In terms of research frontiers, the WOS database focuses more on general stress responses, while the CNKI database is more concerned with specific agricultural and environmental challenges.

To advance future research on “seed-stress” in China, the following recommendations are proposed:

1. Enhance interdisciplinary research: Foster collaboration between disciplines such as plant science, environmental science, and soil science. This interdisciplinary approach can facilitate a comprehensive understanding of the complex mechanisms by which plant seeds respond to environmental stresses, such as the interaction between climate-induced stress and soil-borne stressors affecting seed characteristics.
2. Focus on emerging stress-related topics: Given the increasing complexity of environmental stresses, China should pay closer attention to emerging stress factors and their combined effects on plant seeds. For instance, investigate the impact of novel pollutants or the interaction between multiple stressors like heavy metals and emerging organic pollutants on seed germination, dormancy, and seedling establishment.
3. Deepen research on plant-specific stress-response mechanisms: Conduct in-depth research on the unique stress-response mechanisms of different plant species. Different plants may employ distinct strategies to cope with environmental stresses during the seed stage. Understanding these species-specific mechanisms can provide a foundation for targeted plant conservation and agricultural improvement.
4. Strengthen international collaboration: Actively engage with international research institutions. Through international collaboration, Chinese researchers can access advanced research methodologies and share experiences, keeping pace with the latest international research trends in the field of “seed-stress”. This can also facilitate the exchange of research ideas and data, enabling more comprehensive research.
5. Promote the application of new technologies: Explore the application of emerging technologies, such as gene editing and advanced imaging techniques, in “seed-stress” research. Gene editing can be utilized to modify plant genes associated with stress resistance, while advanced imaging can non-destructively observe internal seed structures and physiological changes under stress, offering new insights into seed-stress responses.

By implementing these strategies, China can elevate its research capabilities in the field of “seed-stress”, contributing to a deeper understanding of plant-environment interactions and providing valuable support for ecological management and conservation.

## Data Availability

The original contributions presented in the study are included in the article/supplementary material. Further inquiries can be directed to the corresponding authors.
